# Organizational justice and long-term metabolic trajectories: a 25-year follow-up of the Whitehall II cohort

**DOI:** 10.1210/clinem/dgab704

**Published:** 2022-01-18

**Authors:** Tibor V. Varga, Tianwei Xu, Mika Kivimäki, Amar J. Mehta, Reiner Rugulies, Naja H. Rod

**Affiliations:** 1 Section of Epidemiology, Department of Public Health, University of Copenhagen, Copenhagen, Denmark; 2 Stress Research Institute, Stockholm University, Stockholm, Sweden; 3 Department of Epidemiology and Public Health, University College London, London, UK; 4 Clinicum, Faculty of Medicine, University of Helsinki, Helsinki, Finland; 5 National Research Centre for the Working Environment, Copenhagen, Denmark; 6 Department of Psychology, University of Copenhagen, Copenhagen, Denmark

**Keywords:** organizational justice, relational justice, metabolic disease, latent cluster analysis, cardiometabolic, trajectory

## Abstract

**Context:**

Organizational justice has been linked to lower risk of several chronic conditions among employees, but less is known about the long-term mechanisms underlying this risk reduction.

**Objective:**

To assess whether self-reported organizational justice is associated with individual and composite metabolic trajectories.

**Design:**

25 years follow-up of the Whitehall II prospective cohort study.

**Setting:**

Middle-aged public servants from the United Kingdom.

**Participants:**

Data on 8,182 participants were used.

**Main outcome measures:**

Levels of eleven anthropometric, glycaemic, lipid and blood pressure biomarkers were measured at five timepoints (1991–2013). We used generalized estimating equations and group-based trajectory modelling to investigate the relationship between organizational justice and biomarker trajectories.

**Results:**

High vs. low organizational justice were associated with lower waist (-1.7 cm) and hip (-1 cm) circumference, BMI (-0.6 kg/m^2^), triglycerides (-1.07 mmol/L) and fasting insulin (-1.08 µIU/mL) trajectories. Two latent metabolic trajectory clusters were identified: a high-risk and a low-risk cluster. High organizational justice (vs. low) were associated with belonging to the low-risk cluster (OR_pooled_=1.47). The low-risk cluster demonstrated lower baseline levels of most biomarkers and better glycaemic control, whereas the high-risk cluster showed higher baseline levels of most biomarkers, glycaemic deterioration, but also greater improvements in lipid levels over time.

**Conclusions:**

People with high organizational justice had more favourable long-term cardiometabolic biomarkers patterns than those with low organizational justice, a potential mechanism contributing to the lower risk of chronic diseases in the first group. Further intervention studies are warranted to determine whether improvement of organizational justice might improve long-term health.

## Introduction

There is longitudinal evidence for associations between work-related stressors, including lack of support, job strain and effort-reward imbalance, and mental(1–3) and cardiometabolic diseases(4–8). From a health prevention perspective, it is equally important to identify resources at the workplace, which can positively impact health. Organizational justice, a measure of how fairly and consistently employees are treated by supervisors, and whether employees feel like they are considered in decision-making processes, is promising in this regard; a high level of organizational justice has previously been associated with better self-rated health(5), better mental health outcomes(9), less inflammation(9), lower risk of persistent insomnia(10), and coronary heart disease(11).

While some mechanisms through which psychosocial factors result in poor health are mapped(12), we still have insufficient understanding of how they contribute to the development of complex metabolic diseases. A number of studies assessed statistical associations between psychosocial work environment factors and metabolic biomarkers(13): many of these biomarkers are believed to be important causal intermediate phenotypes on the pathways towards complex disease outcomes. However, as a recent systematic review on biomarker associations(13) concluded that most of these studies were undertaken in cross-sectional settings and focused on associations between psychosocial stressors and single or few phenotypic outcomes only. The few studies that considered multi-marker profiles most often did so in the framework of investigating the *‘allostatic load’*, which, in simple terms, represents the cumulative metabolic, immunological and endocrine consequences of long-time exposure to chronic stress(14,15). While the overall *‘allostatic load’* concept has been useful for elucidating the link between physiological consequences of chronic stress(16), it arguably reduces the information to a single measure, and ignores the complexity of dynamic interrelationships between metabolites over time, which are only rarely studied(17).

In this study, we set out to assess individual and composite metabolic trajectories of key anthropometric, glycemic, lipid and blood pressure biomarkers over >25 years of the highly characterized Whitehall II study(18). Within this setting, we aim to investigate whether organizational justice is associated with these trajectories. While organizational justice encompasses three main components (distributive, procedural, and relational justice), we focus on the relational component. Through our analysis, we aim to gain insight into different patterns of metabolic changes and systematically describe whether organizational justice might have a role in their progression in midlife and early old age.

## Methods

### The Whitehall II cohort

Between 1985 and 1988, 10,308 nonindustrial civil servants from London, UK were recruited into the Whitehall II prospective cohort study. This population has been extensively studied and recruitment and other protocols are discussed in detail elsewhere(18). In brief, at baseline (Phase 1), a range of sociodemographic, clinical and questionnaire data were collected. Participants at Phase 1 were invited to continue participating in the cohort and were asked to fill in questionnaires and return for clinical examinations in the following years. Of special importance for this study, clinical data on anthropometric, glycaemic, blood pressure and lipid biomarkers were collected at Phase 3 (1991-1993), Phase 5 (1997-1999), Phase 7 (2003-2004), Phase 9 (2008-2009) and Phase 11 (2012-2013) of the study, spanning across >25 years of the participants’ lives.

### Data cleaning and missing data considerations

**Figure 1:**
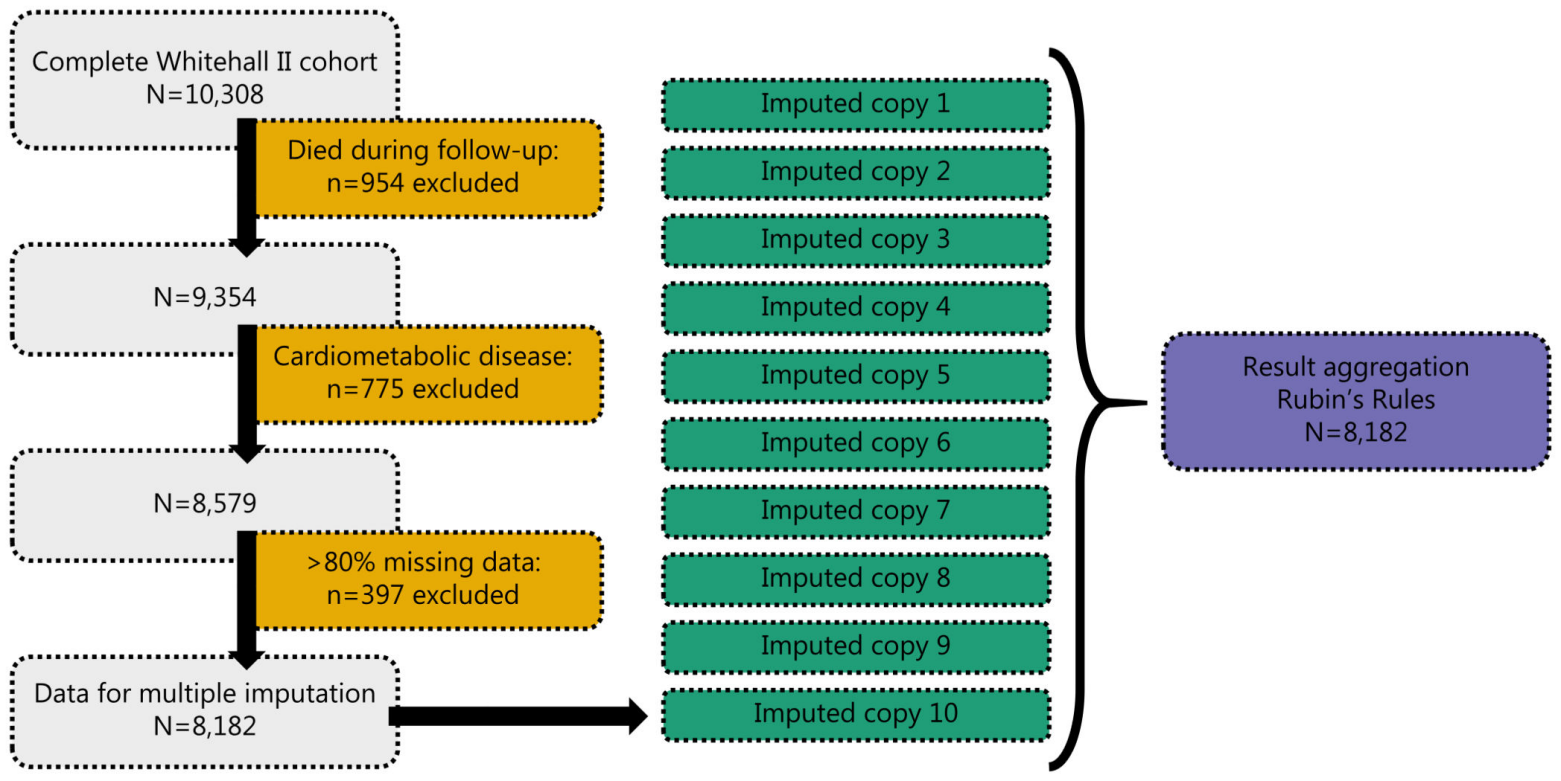
Flowchart of sample selection and multiple imputation framework in the Whitehall II cohort.

The flowchart of sample selection is shown on Figure 1. The original Whitehall II dataset included 10,308 individuals at Phase 1. All individuals who died until Phase 11 (n=954) were excluded; those individuals who were lost to follow-up were kept (n=3,046). From the remaining cohort (n=9,354), individuals with self-reported cardiometabolic illness at baseline (n=775) were excluded. Amongst these individuals, 79 had diabetes and 704 had cardiovascular disease (stroke, heart attack, heart strain, high blood pressure, valve dysfunction, myocardial infarction, or other cardiovascular problems), with some individuals having multiple conditions. Individuals with >80% of their values missing (of all variables) were also removed (n=397) to improve imputation quality.

Missing values were imputed for the remaining cohort of 8,182 individuals using the *missForest* R package(19) in a wide format (variables at different timepoints as separate variables) using default settings of the *missForest()* function. Imputation was undertaken in a multiple imputation framework; the dataset was imputed 10 times and all downstream statistical analyses were repeated in all imputed datasets. Complete case analysis was also performed (n=810).

### Organizational justice

Organizational justice was constructed based on five survey questions at baseline(11): *1. Do you get consistent information from line management (your superior)?; 2. Do you get sufficient information from line management (your superior)?; 3. When you are having difficulties at work, how often is your superior willing to listen to your problems?; 4. Do you ever get criticized unfairly?; 5. Do you ever get praised for your work?* While these questions are primarily indicators of the relational component of organizational justice, the resulting organizational justice scale demonstrated good internal validity in a previous study in the Whitehall II cohort (Cronbach’s alpha=0.71)(5).

Survey responses were collected on 1-4 Likert scales (1=never, 2=seldom, 3=sometimes, 4=often). After imputation (<2% missingness), responses from the five questions were summed into a score ranging between 5-20 (question 4. was reversed). Quartiles were computed and were categorized into *“Low”* (lowest quartile), *“High”* (highest quartile) and *“Intermediate”* (the middle two quartiles) organizational justice.

### Metabolic profiles

Data on eleven metabolic biomarkers were available at five timepoints. The following biomarkers were considered: waist circumference (cm), hip circumference (cm), body mass index (BMI) (kg/m^2^)(20), fasting glucose (mmol/L), fasting insulin (µIU/mL)(21), systolic and diastolic blood pressures (SBP and DBP, respectively) (mmHg)(22), triglycerides, low-density lipoprotein cholesterol (LDL-C), high-density lipoprotein cholesterol (HDL-C) and total cholesterol (TC) (all lipids in mmol/L)(20). Due to skewed distributions, fasting insulin and triglyceride levels were natural log transformed.

### Statistical analysis

All analyses were undertaken using R 4.0.4. and SAS 9.4.

We calculated a correlation matrix of the eleven clinical variables using Pearson correlation. To assess overall correlation between biomarkers, data from all timepoints were used. The statistical relationship between organizational justice at baseline and death during follow-up was assessed using the non-parametric Mann-Whitney test.

Mean trajectories (means and 95% confidence intervals [CIs] at all timepoints) for the eleven biomarkers were calculated for the whole population and stratified by organizational justice. Associations between organizational justice and the trajectories were estimated using generalized estimating equation (GEE) models using the *geepack* R package. The clinical traits were fitted as dependent variables (outcomes) and organizational justice was the independent variable. All models were adjusted for the following potential confounders: age at baseline, sex, ethnicity, educational level, job grade, and income (all self-reported). To assess whether levels of organizational justice were associated with a different slope of change over time in biomarker trajectories, an interaction term of organizational justice and time from baseline was also added to the models. Subsequently, models were repeated by additionally adjusting for potential mediators: marital status, alcohol consumption, smoking status, exercise, diabetes status, presence of any longstanding illness, fasting status and medication use (all self-reported). Ascertainment of potential confounders and mediators has been described previously(20). In GEE model development, we used the *exchangeable* correlation structure, as this produced the lowest Quasi Information Criterion metric from the possible correlation structures (*independent*, *exchangeable*, *first order autoregressive* or *independent*)(23). To obtain sex-specific estimates, GEE analyses were repeated for men and women separately.

Latent classes of multi-marker trajectories were identified using *proc traj* in SAS. After the exclusion of correlated markers (BMI, waist, TG, LDL-C, and DBP, r>0.5), multi-marker trajectories of six traits (the maximum *proc traj* was able to consider) were calculated using a range of settings. Different numbers of latent classes were tested ranging between 1 and 4, with the optimal number of classes chosen based on the lowest Bayesian Information Criterion and Akaike Information Criterion. Linear, quadratic and cubic fit were tested. Model were only considered with class membership >5% and an average posterior probability (a metric of how certainly individuals belong to their cluster on a scale of 0-1) of belonging to a group >0.7, to ensure a high degree of confidence in class membership(24). Metabolic biomarker trajectories, stratified by latent clusters, were visualized and the statistical associations between latent cluster membership and organizational justice were tested using a Pearson’s chi-squared test and odds ratios (OR) were calculated from the contingency tables.

All analyses were undertaken on all imputed datasets (n=10) and results from the imputed datasets were pooled using Rubin’s Rules(25), except for the *P* values from the Pearson’s chi-squared tests, where the median *P* value is presented as previously suggested(26).

## Results

Descriptive statistics are presented in Table 1.

Correlations between metabolic biomarkers across all timepoints are shown in Supplemental Figure 1(27). Strong correlations (|Pearson’s ρ| > 0.6) were observed in three blocks: amongst the three anthropometric variables; SBP and DBP; and LDL-C and TC. We undertook analyses to assess whether organizational justice at baseline was associated with death during follow-up; our results show that those who died have slightly lower levels of organizational justice (on its non-categorized scale of 5-20) compared to those who did not die (15.4 vs 15.6, *P_Mann-Whitney_*=0.014).

### Biomarker trajectories and organizational justice

**Figure 2:**
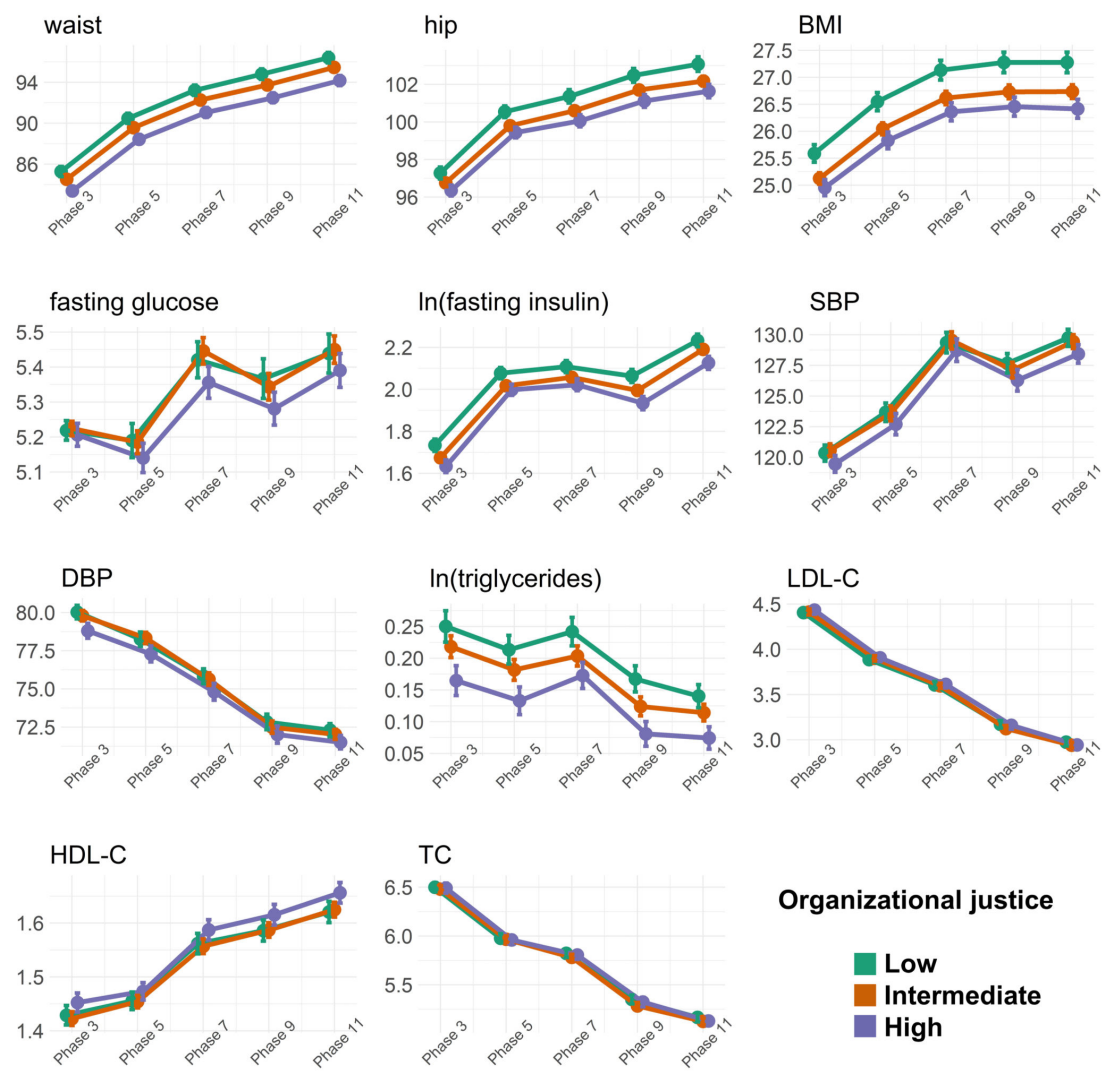
Mean trajectories stratified by levels of organizational justice (N=8,182). The error bars represent 95% confidence intervals of the mean. Abbreviations: BMI – body mass index; DBP – diastolic blood pressure; HDL-C – high-density lipoprotein cholesterol; LDL-C – low-density lipoprotein cholesterol; SBP – systolic blood pressure; TC – total cholesterol

Trajectories stratified by organizational justice for all metabolic traits are shown in Figure 2. Six out of eleven biomarkers worsened over time (waist and hip circumference, BMI, fasting glucose, fasting insulin, SBP), while DBP and lipid levels (triglycerides, LDL-cholesterol, HDL-cholesterol, total cholesterol) improved over time. Individuals with high levels of organizational justice had more beneficial or similar, but never worse, trait levels compared to individuals with low levels of organizational justice, at any timepoint. The magnitude of associations between organizational justice and clinical trait trajectories are shown in Table 2. High vs. low organizational justice was associated with an average of 1.7 cm lower mean waist circumference (95% CI: 1.1; 2.2), a 1.0 cm lower hip circumference (95% CI: 0.6; 1.4), a 0.6 point lower BMI (95% CI: 0.4; 0.9), and lower fasting insulin (β=-1.08 µIU/mL, 95% CI: -1.12; -1.04), DBP (β=-1.1 mmHg, 95% CI: -1.7; -0.5) and triglycerides levels (β=-1.07 mmol/L, 95% CI: -1.11; -1.04). Further adjustment of these models with potential mediators did not materially impact the results (Supplemental Table 1(27)). The interaction terms between organizational justice and time from baseline indicate diverging trajectories for hip and BMI; those with high organizational justice demonstrated slightly less increase in hip circumferences and in BMI over time (hip: β=-0.024 kg/m^2^; 95% CI: -0.04; -0.009, BMI: β=-0.01 kg/m^2^; 95% CI: -0.017; -0.004) compared those with low organizational justice (Supplemental Table 1(27)). The analysis using only those observations with no missing data (n=810) showed estimates consistent with the imputed data, albeit with slightly larger effect sizes for anthropometric traits, fasting insulin, and triglycerides, and no association with DBP (Supplemental Table 2(27)).

Sex-stratified analyses (Supplemental Table 3(27)) revealed that high vs. low organizational justice was associated with an average of 1.4 cm lower mean hip circumference in women (95% CI: -2.3; -0.6), and 0.8 cm lower hip circumference in men (95% CI: -1.2; -0.4). High vs. low organizational justice was also associated with lower fasting insulin, DBP, and TG levels in men. The estimates were directionally consistent in women, but their 95% CIs included unity.

### Latent class analysis of multi-marker trajectories and organizational justice

**Figure 3:**
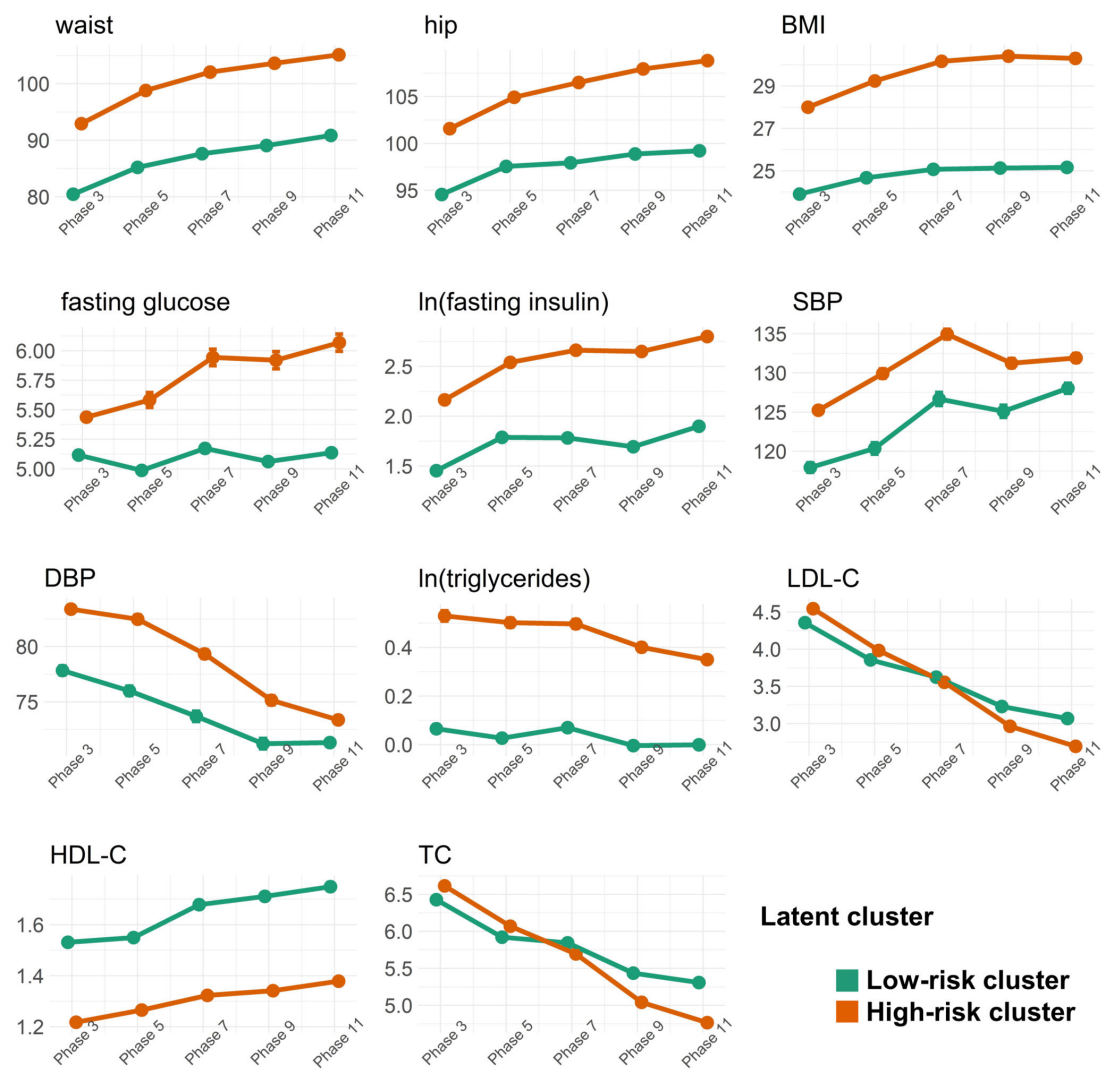
Mean trajectories in the two latent clusters (N=8,182). Latent classes of multi-marker trajectories were identified using *proc traj* in SAS. After the exclusion of correlated markers (BMI, waist, TG, LDL-C, and DBP, r>0.5), multi-marker trajectories of six traits (hip, TC, HDL-C, SBP, fasting glucose, and fasting insulin) were calculated. The clusters identified and presented on this plot were obtained by considering two clusters and a linear fit. The error bars represent 95% confidence intervals of the mean. Abbreviations: BMI – body mass index; DBP – diastolic blood pressure; HDL-C – high-density lipoprotein cholesterol; LDL-C – low-density lipoprotein cholesterol; SBP – systolic blood pressure; TC – total cholesterol.

We also combined information from all metabolic biomarkers into multi-dimensional trajectories, and a comparison of clustering approaches with 1 to 4 latent groups are shown in Supplemental Table 4(27). One latent class was the best fit for the data (Supplemental Text 1(27)). However, we also visualized the two-cluster linear fit (Figure 3) and the three-cluster linear fit (Supplemental Text 2(27)) for all clinical biomarkers. The two-cluster fit was the most informative and clinically interpretable, so we further analysed these clusters. The low-risk metabolic cluster comprised 69% of the population (posterior probability=98%). The high-risk metabolic cluster comprised 31% of the population (posterior probability=97%). The low risk cluster is, compared to the high risk cluster, characterized by more beneficial levels of nine of the 11 biomarkers. The exceptions were LDL cholesterol and total cholesterol, where the values were similar in the low-risk and high risk cluster. Means and 95% CIs of biomarker levels in the two cluster are shown in Supplemental Table 5(27). For fasting glucose, the low-risk metabolic cluster demonstrates a constant average over time (horizontal trajectory), whereas the high-risk cluster shows higher baseline levels and glycaemic deterioration over time, but greater improvement in lipid levels, especially for LDL-C and TC, over the follow-up period. We further investigated whether there are differences in rates of prescribed medications for cardiovascular diseases over time between the two clusters, and observed differences at all five timepoints with higher percentages of the high-risk cluster (1.4x-2.3x across all timepoints) taking cardiovascular medications (Supplemental Text 3(27)).

**Figure 4:**
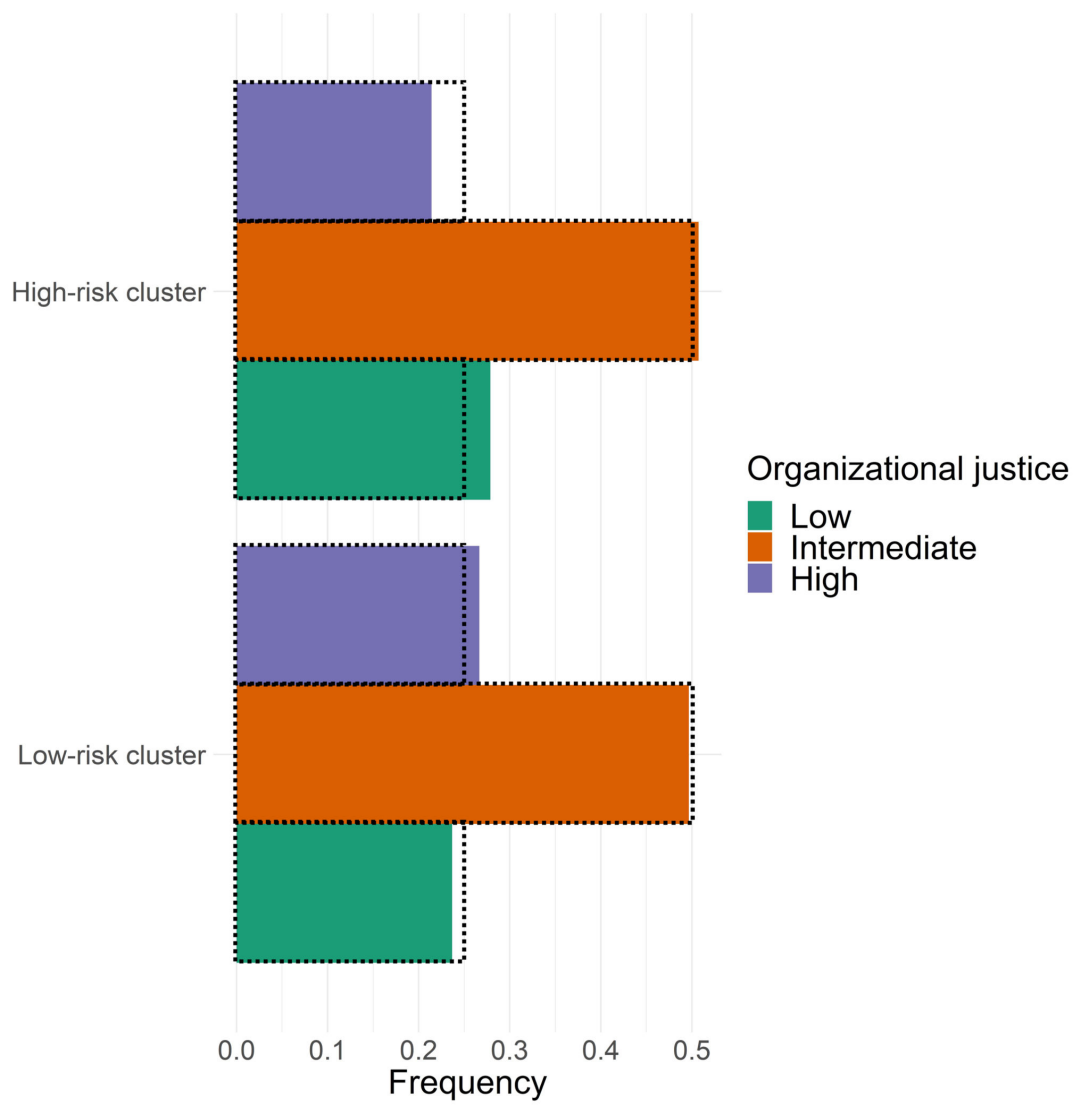
Cluster membership and organizational justice (N=8,182). Latent classes of multi-marker trajectories were identified using *proc traj* in SAS. After the exclusion of correlated markers (BMI, waist, TG, LDL-C, and DBP, r>0.5), multi-marker trajectories of six traits (hip, TC, HDL-C, SBP, fasting glucose, and fasting insulin) were calculated. The clusters identified and presented on this plot were obtained by considering two clusters and a linear fit. The dotted line represents the expected frequencies for low organizational justice (25%), intermediate organizational justice (50%), and high organizational justice (25%), while the colour-filled bars represent the observed frequencies of these categories in the High-risk and Low-risk clusters (pooled OR=1.47; *P_chi-squared_*=6.1×10^-8^).

As shown in Figure 4, the low-risk cluster shows an overrepresentation of individuals with high organizational justice (27% vs. the expected 25% of the population), while the high-risk cluster shows an overrepresentation of individuals with low organizational justice (28% vs. the expected 25% of the population). Thus, we reject the null hypothesis that cluster membership and levels of organizational justice are independent (OR = 1.47, *P*=6.1×10^-8^).

## Discussion

This study shows that there is an inverse relationship between organizational justice, and long-term metabolic trajectories of a number of anthropometric, glycaemic and lipid biomarkers. Moreover, organizational justice also associates with the latent clusters of composite metabolic trajectories that appear to divide the population into a high- and low-risk metabolic trajectory profile.

While organizational justice has been linked to various health outcomes(5,9,11), this is the first study to investigate how it relates to long-term metabolic trajectories spanning across 25 years with eleven biomarkers. Over time, the study population experienced a deterioration in all anthropometric and glycaemic traits and SBP levels on average. However, DBP levels and lipid profiles improved over time, most likely due to the adaption of healthier lifestyles and the widespread use of lipid-lowering medications, as suggested previously(28). The simultaneous increase in SBP and decrease in DBP suggest increasing average pulse pressure in the population, which is a marker for deteriorating vascular elasticity, robustly linked to cardiovascular disease(29). Those who experienced higher organizational justice demonstrated better metabolic profiles from the beginning of the study period with lower levels of waist, hip, BMI, fasting insulin, and triglyceride levels. As an example, the 1.7 cm difference in waist circumference observed between those with high and low organizational justice is comparable in magnitude to a waist increase observed over ~5 years in this cohort on average. Furthermore, over time, individuals with high organizational justice also showed a slower deterioration in their waist, hip and BMI levels compared to those with low organizational justice. These observations were consistent with those observed in the sex-stratified analyses. We observed a larger difference in anthropometric traits level changes between those with high and low levels of organizations justice in women than in men. However, the stratified analyses were statistically underpowered to exhaustively investigate possible risk factors that may explain differences in associations by sex. Menopause and other sex-specific health-related factors are likely to be important confounders, potentially playing dual roles in impacting work-related psychosocial health (30,31) and affecting physical health, e.g. via deteriorations in bone health and lipid profiles (32), and thus warrant future research.

There are multiple pathways through which organizational justice might have exerted an effect on metabolic trajectories. First, organizational injustices are likely to result in poor mental health outcomes (e.g. stress, anxiety and depression), which, through neurological and endocrine pathways, or even through alterations in the gut microbiome, have been linked to chronic metabolic deterioration(33,34). Second, injustices at the workplace might result in adapting unhealthful coping mechanisms (e.g. smoking)(35,36) or other negative lifestyle changes that could, in turn, negatively impact metabolic outcomes(37). It is an interesting finding that organizational justice is primarily related to elevations in metabolic factors that characterize an insulin-resistant phenotype with higher levels of adiposity, fasting insulin and triglyceride levels, but show no evidence or only suggestive associations with markers more proximal to cardiovascular outcomes, such as high blood pressure and LDL-C and HDL-C levels. These findings can be juxtaposed to previously detected associations between job strain and effort-reward imbalance and coronary heart disease and stroke, hypothesized to be primarily linked by alterations in blood pressure and lipid levels(38,39). As differences in blood pressure and LDL-C and HDL-C trajectories were much smaller between groups defined by high and low levels of organizational justice in this study, our results might cautiously indicate a distinct pathway for how organizational justice might relate to long-term metabolic changes and disease incidence. However, further studies are needed to confirm or refute this hypothesis.

We identified two latent clusters in the Whitehall II cohort. The low-risk cluster is characterized by remarkably good glycaemic control over the entire follow-up period on average, with fasting glucose levels remaining around their baseline levels, and fasting insulin levels showing a less steep deterioration compared to the other cluster. Individuals in this low-risk cluster also showed more beneficial blood pressure values and were leaner on average compared to those in the high-risk cluster. Individuals in the high-risk cluster, albeit worse overall metabolic profiles, demonstrated greater improvements in their lipid levels compared to the low-risk cluster to the extent that average levels of LDL-C and TC were actually lower for this group at the final timepoint compared to the low-risk cluster. This greater improvement in lipid levels in the high-risk cluster is likely to be attributed to the fact that individuals here were more commonly prescribed drugs for cardiovascular diseases compared to those in the low-risk cluster; this is also reflected in improving blood pressure levels observed over the last two timepoints. As commonly prescribed statins have been linked to increasing fasting glucose levels in individuals both with or without diabetes(40), imbalances in taking these medications between the two clusters could be a contributing factor to the differences in glycaemic deterioration that we observe. We also found evidence for a slight overrepresentation of individuals with high organizational justice in the low-risk cluster, confirming our observations from the individual biomarker trajectories.

The main strengths of this study are the large sample size, the long follow-up with multiple measurement points, and the use of long-term trait-specific and composite metabolic trajectories. The detailed phenotypic measurements at multiple timepoints offer an excellent opportunity to study long-term biomarker trajectories. The use of a robust multiple imputation framework, the advanced statistical methods to assess statistical associations between trajectories, and the data-driven identification of latent clusters also represent important strengths.

The most important limitation of this study is that organizational justice has been collected only at baseline, whereas the outcomes were collected at later timepoints, up until 25 years after baseline, where levels of organizational justice may have changed. While our findings emanate from a prospective setting, it is not possible to establish whether the association between organizational justice and the metabolic outcomes are causal in nature. Missing values, especially those due to loss to follow-up, represent a challenge that we addressed using multiple imputation. In this framework, those missing values with more uncertainty will vary more across imputed datasets, and results from all imputed copies are subsequently pooled. Multiple imputation strategies have been shown to be a suitable alternative to other commonly used approaches to address loss to follow-up, such as inverse probability weighting(41,42). In line with this consideration, although we performed complete case analysis, we believe that these results are prone to selection bias. As reported previously, loss to follow-up in this population is slightly higher amongst those with lower levels of organizational justice at baseline(5); our additional analysis shows that those who died during follow-up have slightly lower levels of organizational justice on average at baseline. These phenomena might bias our findings in a way that our observed associations actually underestimate true relationships. One latent class was the best fit for our data; this observation indicates homogeneity in the cohort and it is likely that larger sample sizes would have resulted in more statistical power to identify more latent clusters in our data. However, we believe that the investigation of the two latent clusters yielded interesting, and clinically relevant results.

As it has been described previously, although a robust proxy for organizational justice, the survey questions we used in this study mainly measure relational justice, an aspect that relates to interpersonal aspects of fairness (i.e. whether supervisors are fair and consistent to employees at the workplace, cooperative behaviour, or dialogue among actors in a post-conflict situation)(5,43). While organizational justice is believed to be an important determinant of employee health(5), it is possible that the observed trajectory differences are not fully attributable to this particular factor, but also relate to a number of other positive and negative psychosocial work environment factors, that are often strong correlates of organizational justice. As socioeconomic determinants are root causes of health and disease(44), it is also likely that socioeconomic confounders impacted our results. We aimed to mitigate this by adjusting for educational attainment, annual income, and job grade in all our statistical models. Organizational justice, and a number of other confounders and mediators are self-reported; this type of data is prone to bias, and potentially results in inflated statistical associations. Some of the biomarkers used in this study are sensitive to fasting status and other daily fluctuations; we aimed to offset this risk by adjusting for fasting status and underlying disease conditions and our results show negligible influence of these adjustments.

Our study adds important insights to the body of evidence linking psychosocial work environment factors and health outcomes, as it demonstrates a relationship between organizational justice and individual and complex multi-marker metabolic trajectories, which are likely conduits in the development of metabolic disease outcomes, such as type 2 diabetes and other cardiometabolic diseases.

## Supplementary Material

Figure S1

STROBE checklist

Table S1

Table S2

Table S3

Table S4

Table S5

Text S1

Text S2

Text S3

## Figures and Tables

**Table 1:** Descriptive statistics of Whitehall II participants analysed in this study (N=8,182).

	Phase 3	Phase 5	Phase 7	Phase 9	Phase 11
**sex (women / men)**	2,650 (32%) / 5,532 (68%)				
**ethnicity (white / non-white)**	7,411 (91%) / 771 (9%)				
**educational attainment (up to 16 yrs / until 17-18 yrs / over 18 yrs)**	2,700 (33%) / 1,650 (20%) / 3,832 (47%)				
**income (<19999 GBP / 20000-39999 GBP / 40000-59999 GBP / >60000 GBP)**	2,169 (26%) / 3,280 (40%) / 1,614 (20%) / 1,119 (14%)				
**job grade (administrative / prof & exec / clerical & support)**	2,451 (30%) / 4,026 (49%) / 1,705 (21%)				
**baseline age (yrs)**	44.4 ± 5.9				
**organizational justice scale (5-20)**	15.8 ± 2.5				
**waist (cm)**	84.4 ± 11.21	89.5 ± 11.15	92.2 ± 11.47	93.7 ± 11.45	95.4 ± 11.65
**hip (cm)**	96.8 ± 7.01	99.9 ± 7.00	100.7 ± 7.90	101.8 ± 8.25	102.3 ± 8.66
**BMI (kg/m^2^)**	25.2 ± 3.60	26.1 ± 3.75	26.7 ± 4.08	26.8 ± 4.24	26.8 ± 4.23
**fasting glucose (mmol/L)**	5.2 ± 0.78	5.2 ± 1.11	5.4 ± 1.24	5.3 ± 1.26	5.4 ± 1.30
**ln(fasting insulin) (ln(uIU/mL))**	1.7 ± 0.77	2.0 ± 0.67	2.1 ± 0.73	2.0 ± 0.81	2.2 ± 0.80
**SBP (mmHg)**	120.2 ± 20.6	123.4 ± 24.5	129.3 ± 28.2	127.0 ± 26.5	129.3 ± 21.7
**DBP (mmHg)**	79.6 ± 13.91	78.1 ± 14.39	75.5 ± 16.13	72.5 ± 16.76	72.0 ± 12.92
**ln(triglycerides) (ln(mmol/L))**	0.2 ± 0.60	0.2 ± 0.57	0.2 ± 0.56	0.1 ± 0.52	0.1 ± 0.46
**LDL-C (mmol/L)**	4.4 ± 1.16	3.9 ± 1.06	3.6 ± 1.00	3.1 ± 1.18	2.9 ± 1.21
**HDL-C (mmol/L)**	1.4 ± 0.44	1.5 ± 0.40	1.6 ± 0.48	1.6 ± 0.48	1.6 ± 0.49
**TC (mmol/L)**	6.5 ± 1.32	6.0 ± 1.26	5.8 ± 1.14	5.3 ± 1.26	5.1 ± 1.24

For categorical variables, counts and percentages of total population are presented. For continuous traits, means and standard deviations are presented. Abbreviations: BMI – body mass index; DBP – diastolic blood pressure; GBP - British pound sterling; HDL-C – high-density lipoprotein cholesterol; LDL-C – low-density lipoprotein cholesterol; SBP – systolic blood pressure; TC – total cholesterol

**Table 2:** Associations between organizational justice and biomarker trajectories among 8,182 participants in the Whitehall II study.

Outcome trajectory	High vs. low organizational justice Main effect	Intermediate vs. low organizational justice Main effect
waist (cm)	-1.7 (-2.2; -1.1)	-0.1 (-1.5; -0.5)
hip (cm)	-1.0 (-1.4; -0.6)	-0.5 (-0.9; -0.2)
BMI (kg/m^2^)	-0.6 (-0.9; -0.4)	-0.4 (-0.6; -0.2)
fasting glucose (mmol/L)	-0.02 (-0.07; 0.03)	0.01 (-0.03; 0.05)
ln(fasting insulin) (ln(µIU/mL))	-0.08 (-0.11; -0.04)	-0.05 (-0.08; -0.02)
SBP (mmHg)	-0.8 (-1.65; 0.06)	0.4 (-0.4; 1.1)
DBP (mmHg)	-1.1 (-1.7; -0.5)	-0.1 (-0.6; 0.4)
ln(triglycerides) (ln(mmol/L))	-0.07 (-0.1; -0.04)	-0.03 (-0.06; -0.002)
LDL-C (mmol/L)	0.03 (-0.03; 0.1)	0.02 (-0.03; 0.07)
HDL-C (mmol/L)	0.002 (-0.02; 0.02)	-0.01 (-0.02; 0.01)
TC (mmol/L)	-0.02 (-0.09; 0.05)	-0.01 (-0.07; 0.05)

Covariates: organizational justice × time, age at baseline, sex, ethnicity, educational level, job grade, income. The estimates are presented with 95% confidence intervals. Abbreviations: BMI – body mass index; DBP – diastolic blood pressure; HDL-C – high-density lipoprotein cholesterol; LDL-C – low-density lipoprotein cholesterol; SBP – systolic blood pressure; TC – total cholesterol

## Data Availability

Individual level data from the Whitehall II cohort cannot be shared due to ethical reasons. However, data can be obtained through a formal data request process, as outlined here: https://www.ucl.ac.uk/epidemiology-health-care/research/epidemiology-and-public-health/research/whitehall-ii/data-sharing
