# Supplementary figures and images for: Organizational justice and long-term metabolic trajectories: a 25-year follow-up of the Whitehall II cohort
Source: J Clin Endocrinol Metab. Author manuscript; Available in PMC 2022 Apr 26. (PMC8764354; doi:10.1210/clinem/dgab704)

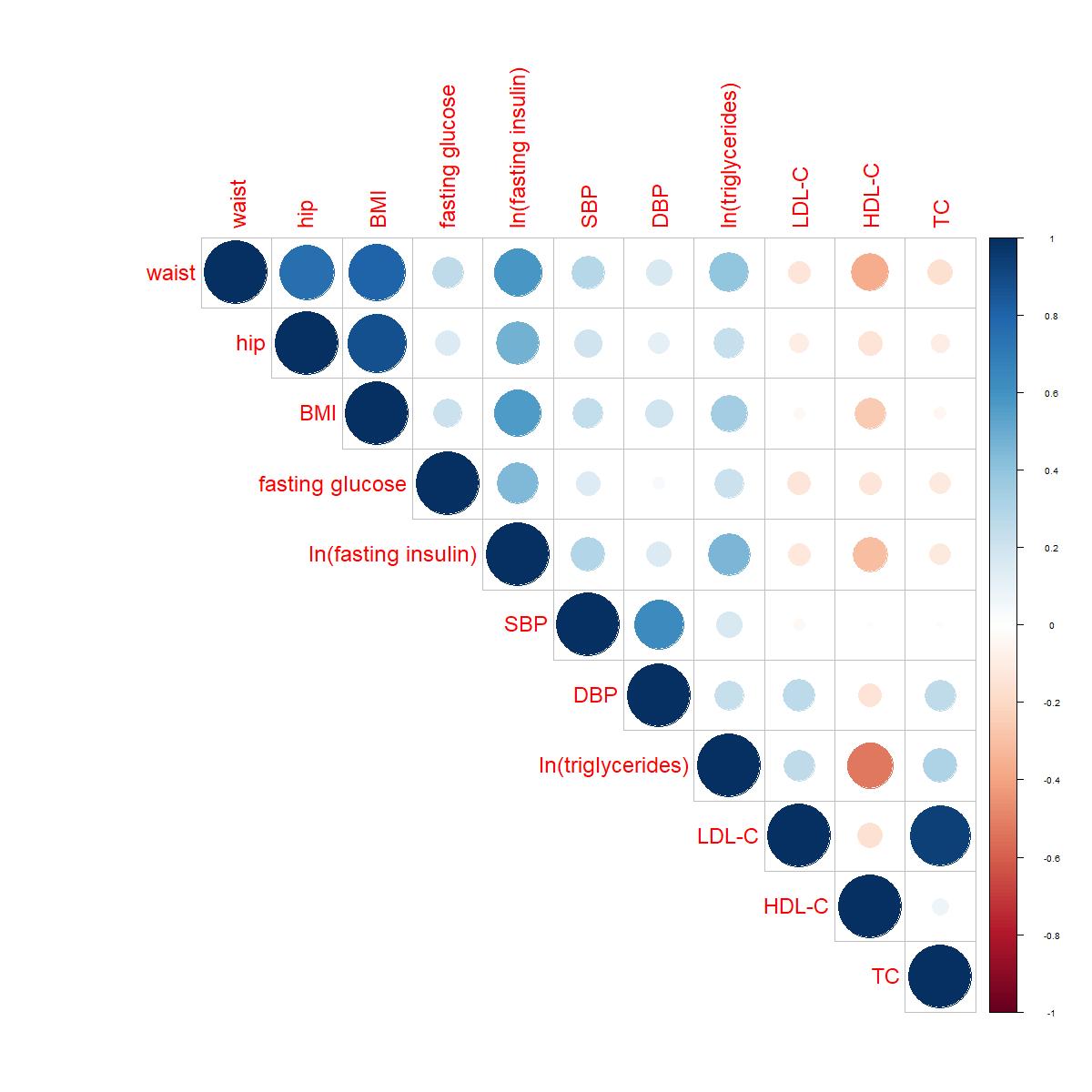

Supplement: Figure S1 [file EMS136013-supplement-Figure_S1.jpg]
