# Supplementary material for: Organizational justice and long-term metabolic trajectories: a 25-year follow-up of the Whitehall II cohort
Source: J Clin Endocrinol Metab. Author manuscript; Available in PMC 2022 Apr 26. (PMC8764354; doi:10.1210/clinem/dgab704)

**Supplemental Text 1**. Biomarker trajectories in one latent cluster (i.e. the whole population) (N=8,182).

Mean values and standard errors for the eleven biomarkers were pooled from the ten imputed datasets, at all timepoints, using Rubin’s Rules. 95% confidence intervals were calculated and visualized.


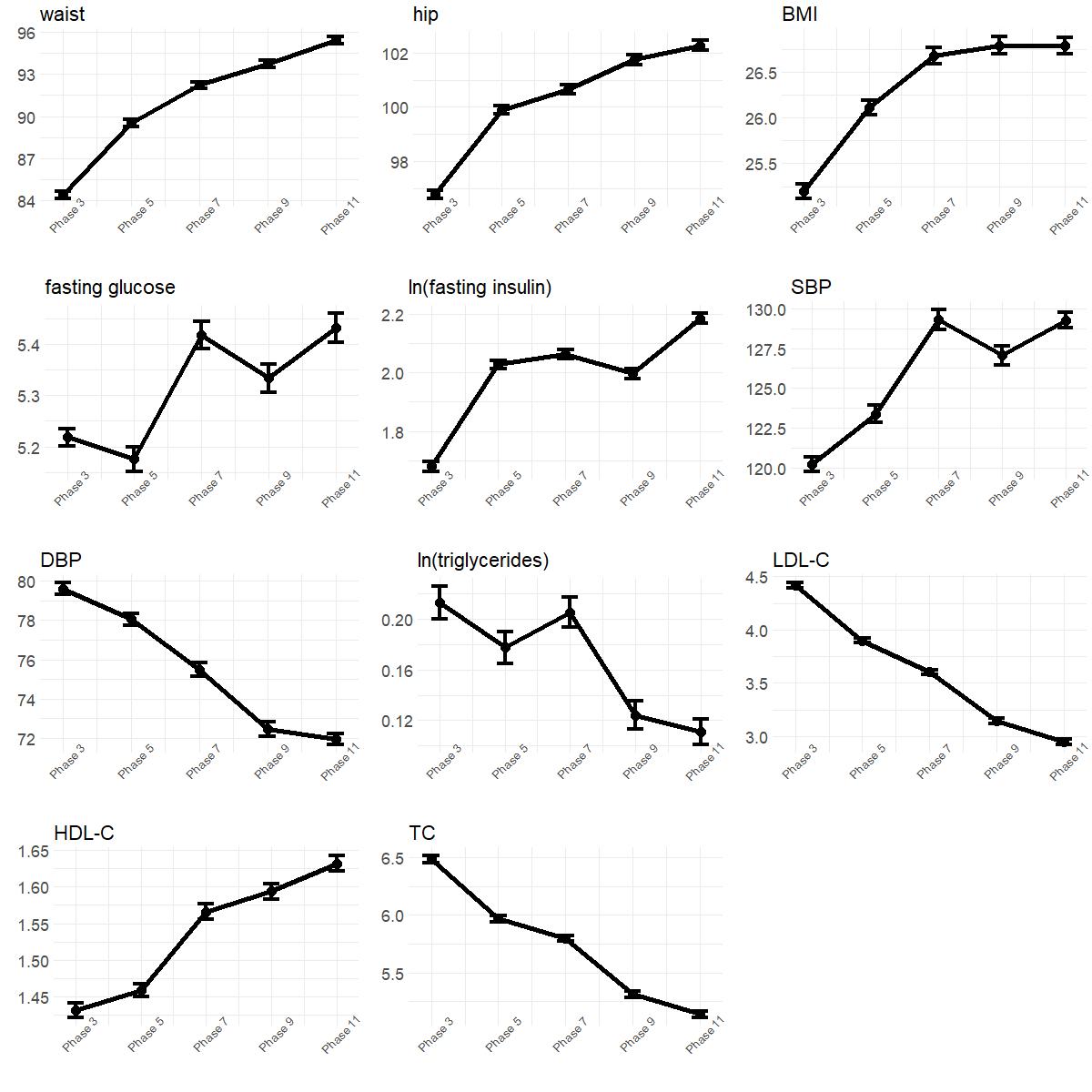

Supplement: Text S1 [file EMS136013-supplement-Text_S1.docx]
