# Supplementary material for: Organizational justice and long-term metabolic trajectories: a 25-year follow-up of the Whitehall II cohort
Source: J Clin Endocrinol Metab. Author manuscript; Available in PMC 2022 Apr 26. (PMC8764354; doi:10.1210/clinem/dgab704)

**Supplemental Text 2**. Biomarker trajectories in the three latent clusters (N=8,182).

Due to the multiple imputation framework, the three clusters were reproduced in six out of the ten imputed datasets. Mean values and standard errors for the eleven biomarkers were pooled from the six imputed datasets, at all timepoints, using Rubin’s Rules. 95% confidence intervals were calculated and visualized.


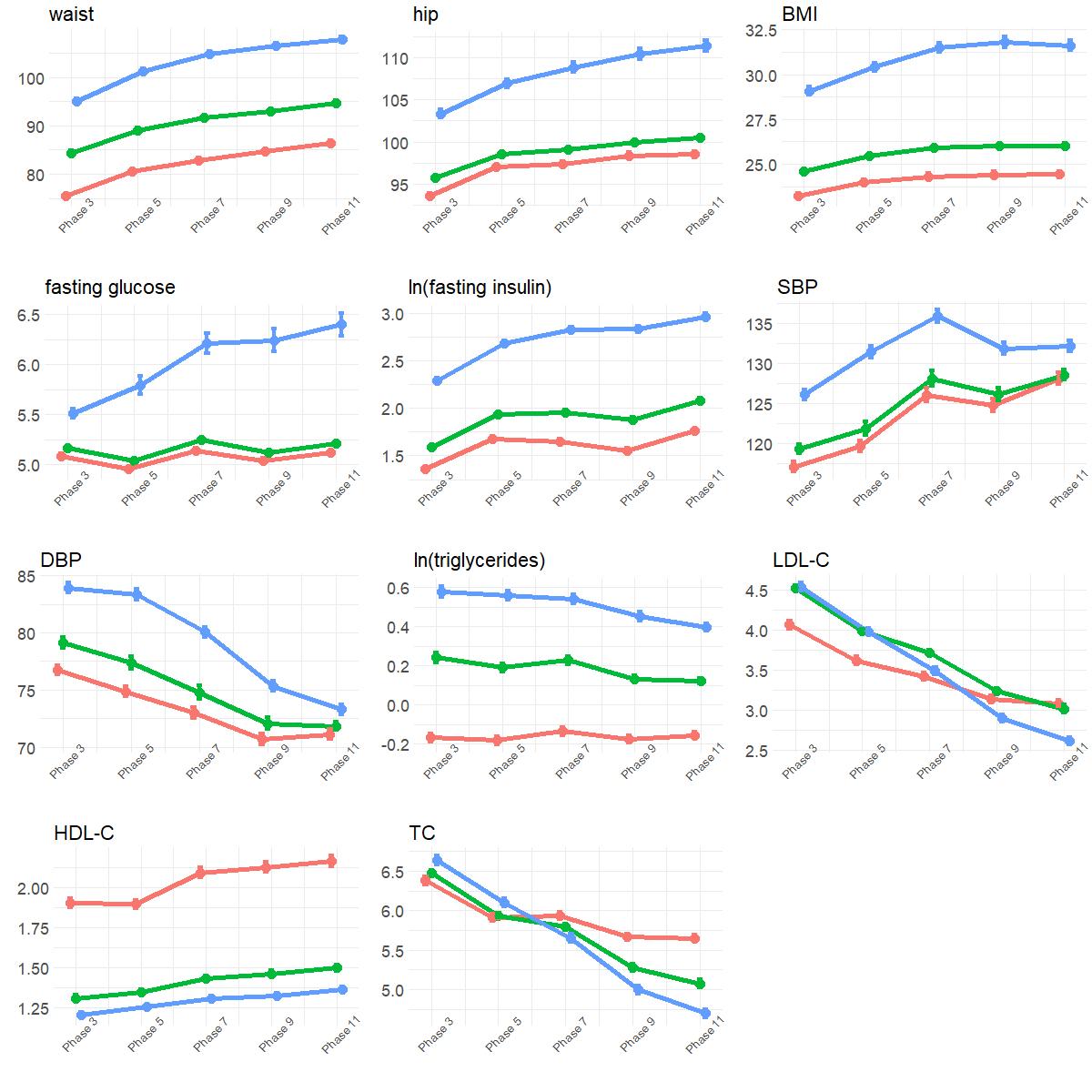

Supplement: Text S2 [file EMS136013-supplement-Text_S2.docx]
