# Supplementary material for: Organizational justice and long-term metabolic trajectories: a 25-year follow-up of the Whitehall II cohort
Source: J Clin Endocrinol Metab. Author manuscript; Available in PMC 2022 Apr 26. (PMC8764354; doi:10.1210/clinem/dgab704)

**Supplemental Text 3**. Latent clusters and cardiovascular medication (N=8,182).

We utilized chi-squared tests to investigate whether latent cluster membership (high-risk vs. low-risk cluster) is independent from taking drugs for cardiovascular problems (yes vs. no). We undertook this analysis at all five timepoints (Phases 3, 5, 7, 9 & 11).

**Phase 3**

| *Cluster membership* | *Drug* | *Percentage* |
| --- | --- | --- |
| *Low-risk* | *No* | *96%* |
|  | *Yes* | *4%* |
| *High-risk* | *No* | *80%* |
|  | *Yes* | *10%* |

*P_chi-squared_* = 2.7×10^-24^


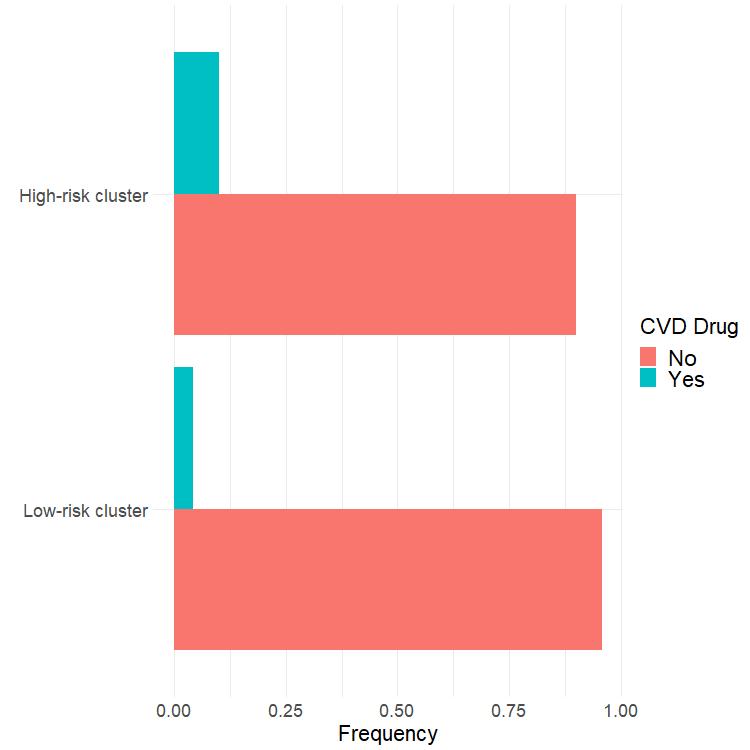


**Phase 5**

| *Cluster membership* | *Drug* | *Percentage* |
| --- | --- | --- |
| *Low-risk* | *No* | *91%* |
|  | *Yes* | *9%* |
| *High-risk* | *No* | *80%* |
|  | *Yes* | *20%* |

*P_chi-squared_* = 1.9×10^-47^


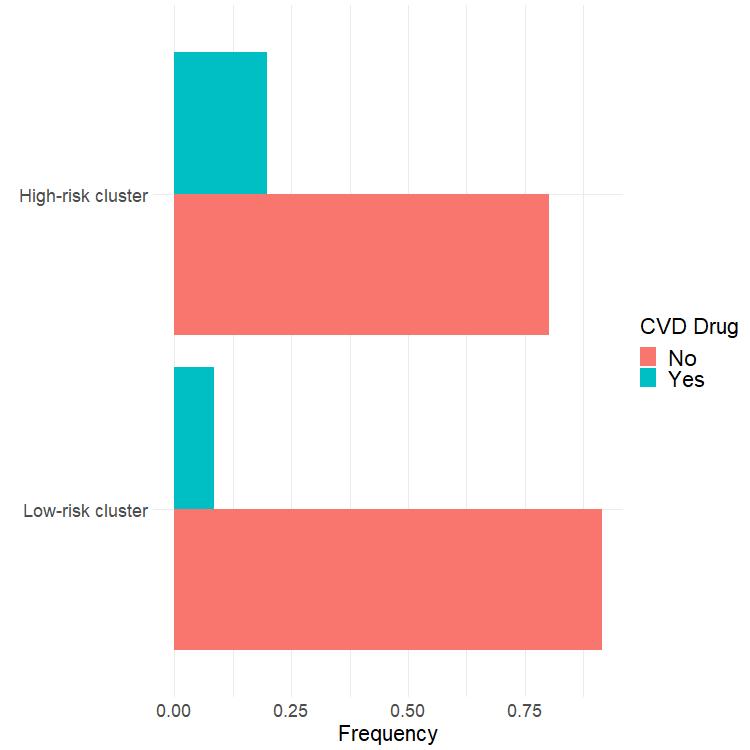


**Phase 7**

| *Cluster membership* | *Drug* | *Percentage* |
| --- | --- | --- |
| *Low-risk* | *No* | *83%* |
|  | *Yes* | *17%* |
| *High-risk* | *No* | *63%* |
|  | *Yes* | *37%* |

*P_chi-squared_* = 1.1×10^-87^


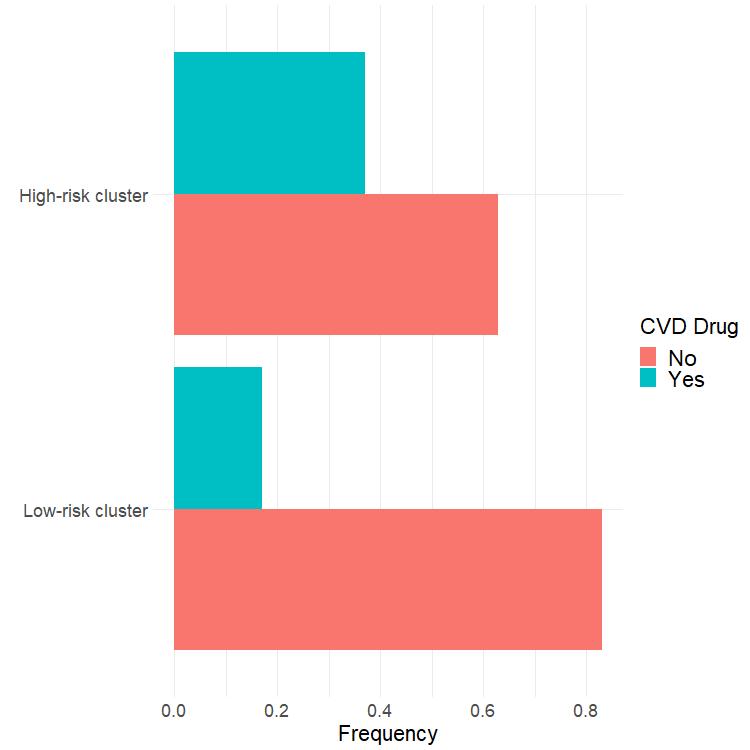


**Phase 9**

| *Cluster membership* | *Drug* | *Percentage* |
| --- | --- | --- |
| *Low-risk* | *No* | *55%* |
|  | *Yes* | *45%* |
| *High-risk* | *No* | *32%* |
|  | *Yes* | *68%* |

*P_chi-squared_* = 2.6×10^-80^


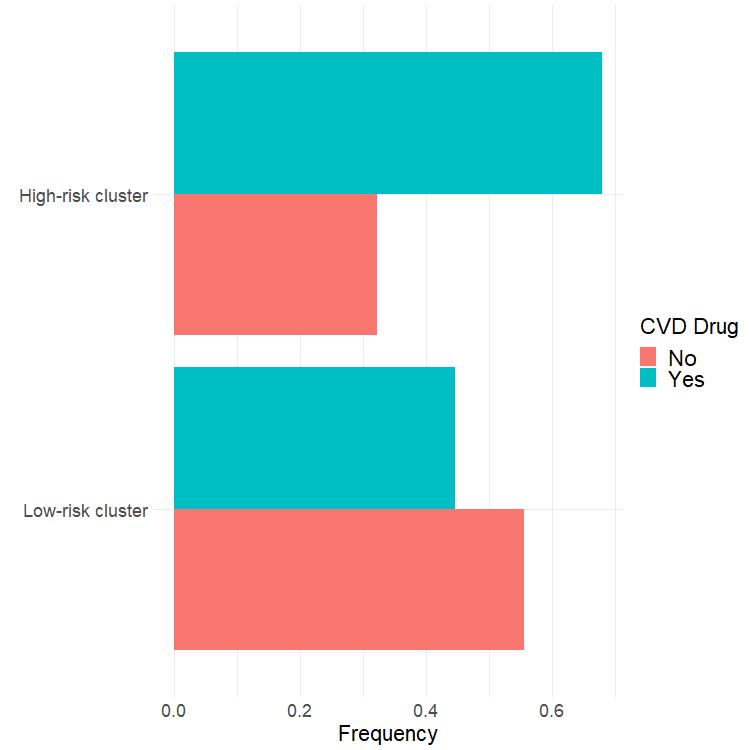


**Phase 11**

| *Cluster membership* | *Drug* | *Percentage* |
| --- | --- | --- |
| *Low-risk* | *No* | *47%* |
|  | *Yes* | *53%* |
| *High-risk* | *No* | *23%* |
|  | *Yes* | *77%* |

*P_chi-squared_* = 3.1×10^-90^


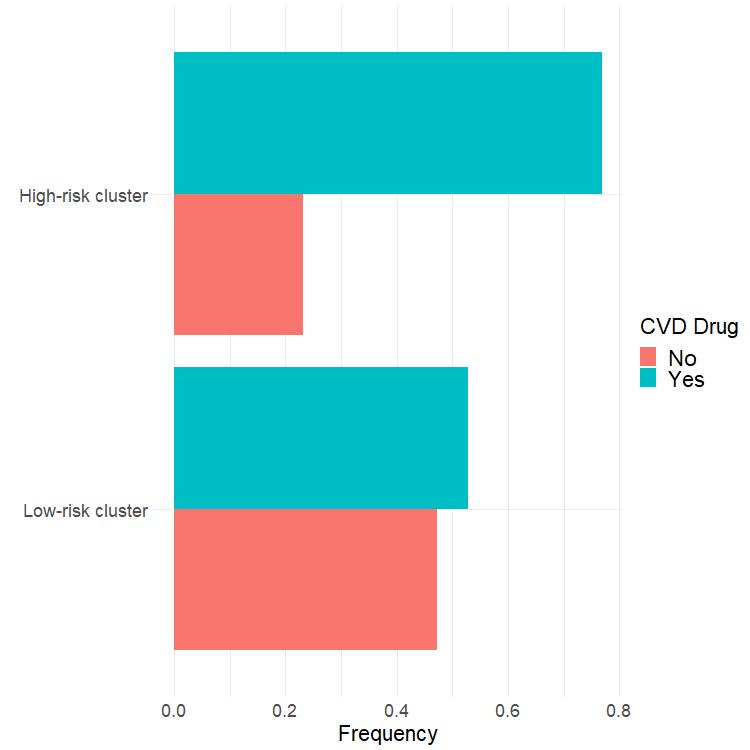

Supplement: Text S3 [file EMS136013-supplement-Text_S3.docx]
